# Epigenetic readers and lung cancer: the rs2427964C>T variant of the bromodomain and extraterminal domain gene *BRD3* is associated with poorer survival outcome in NSCLC

**DOI:** 10.1002/1878-0261.13109

**Published:** 2021-10-15

**Authors:** Jang Hyuck Lee, Seung Soo Yoo, Mi Jeong Hong, Jin Eun Choi, Hyo‐Gyoung Kang, Sook Kyung Do, Won Kee Lee, Sun Ha Choi, Yong Hoon Lee, Hyewon Seo, Jaehee Lee, Shin Yup Lee, Seung Ick Cha, Chang Ho Kim, Eung Bae Lee, Sukki Cho, Sanghoon Jheon, Jae Yong Park

**Affiliations:** ^1^ Department of Biochemistry and Cell Biology School of Medicine Kyungpook National University Daegu Korea; ^2^ Cell and Matrix Research Institute School of Medicine Kyungpook National University Daegu Korea; ^3^ Department of Internal Medicine School of Medicine Kyungpook National University Kyungpook National University Hospital Daegu Korea; ^4^ Biostatistics, Medical Research Collaboration Center Kyungpook National University Daegu Korea; ^5^ Department of Thoracic Surgery School of Medicine Kyungpook National University Daegu Korea; ^6^ Department of Thoracic and Cardiovascular Surgery School of Medicine Seoul National University Korea; ^7^ BK21 Plus KNU Biomedical Convergence Program Department of Biomedical Science Kyungpook National University Daegu Korea

**Keywords:** BET genes, epigenetics, lung cancer, polymorphisms, prognosis

## Abstract

Bromodomain and extraterminal domain (BET) proteins are epigenetic readers that regulate gene expression. We investigated whether variants in BET genes are associated with survival outcomes for lung cancer. To do this, the associations between 77 variants in BET family genes and survival outcomes were analyzed in 773 non‐small‐cell lung cancer (NSCLC) patients who underwent surgery (349 and 424 patients in the discovery and validation cohorts, respectively). We found that six variants were significantly associated with overall survival (OS) in the discovery cohort, and one variant (rs2506711C>T) was replicated in the validation cohort. *BRD3* rs2506711C>T is located in the repressed area and has a strong linkage disequilibrium with rs2427964C>T in the promoter region. *BRD3* rs2427964C>T was significantly associated with worse OS in the discovery cohort, validation cohort, and combined analysis. In a luciferase assay, promoter activity in the *BRD3* rs2427964 T allele was significantly higher than that in the *BRD3* rs2427964 C allele, which selectively bound with the transcriptional repressor SIN3A. Knockdown of *BRD3* with *BRD3*‐specific siRNA decreased the proliferation and migration of lung cancer cells while also increasing the rate of apoptosis. These results suggest that *BRD3* rs2427964C>T increases *BRD3* expression through increased promoter activity, which is associated with poor prognosis for lung cancer.

## Graphical abstract

In this study, we investigated the association of *BET* gene variants with survival of patients with non‐small‐cell lung cancer (NSCLC). We observed that the rs2427964C>T SNP in the *BRD3* promoter region was associated with poorer survival outcome. *BRD3* promoter activity was higher in rs2427964_T than in rs2427964_C, which selectively bound with the transcriptional repressor SIN3A. Additionally, *BRD3* silencing decreased the proliferation and migration of NSCLC cells. Our data suggest that elevated *BRD3* expression regulated by rs2427964C>T leads to reduced overall survival in patients with NSCLC.

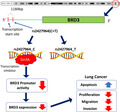

**Abbreviations**
aHR: adjusted hazard ratio
BET: bromodomain and extraterminal domain
BRD: bromodomain
CIs: confidence intervals
CTCF: CCCTC‐binding factor
LD: linkage disequilibrium
MAF: minor allele frequency
NSCLC: non‐small‐cell lung cancer
OS: overall survival
rSNP: regulatory single nucleotide polymorphisms

## 1 Introduction

Although cancer is generally considered a genetic disease, epigenetic aberration also plays an important role in carcinogenesis [1]. DNA methylation, histone modification, and microRNA gene silencing are examples of epigenetic modifications that can alter gene expression without changing nucleotide sequences. Recent studies have shown that epigenetic abnormalities are commonly found in human cancers and are involved in almost all stages of cancer development [1, 2]. For example, histone modifications, such as methylation, phosphorylation, and acetylation, are necessary for gene expression. Aberrations in histone modification are associated with carcinogenesis or the prognosis of several cancer types including lung cancer [3, 4]. Barlési *et al*. [5] found that epigenetic changes in histone modifications were significantly associated with disease‐free and overall survival (OS) of resected non‐small‐cell lung cancer (NSCLC). In addition, Van Den Broeck *et al*. [6] reported that the loss of histone H4K20 trimethylation influenced NSCLC prognosis.

Single nucleotide polymorphisms (SNPs) are the most common type of genetic variation; they occur on average almost once every 1000 nucleotides. Most SNPs do not affect health or disease, but a small number of have a significant impact on disease development or prognosis. In particular, when a SNP occurs in a regulatory region within or near a gene, it may affect the function of the gene, which increases the likelihood of affecting disease. Regulatory single nucleotide polymorphisms (rSNPs) are variants of transcription factor binding sites within gene promoter regions or enhancers; rSNPs are known to alter gene expression and affect human disease [7, 8].

In the histone modification pathway, bromodomain and extraterminal domain (BET) proteins serve as epigenetic readers that regulate gene expression by recruiting chromatin‐regulating enzymes of histone modification [9]. The BET family of proteins is composed of bromodomain‐containing (BRD)2, BRD3, BRD4, and bromodomain testis‐specific protein [10]. Dysregulation of BET family genes has been reported in cancers [11, 12, 13]. BET inhibitors targeting BET proteins have also been investigated; the BET inhibitors JQ1 and I‐BET151 are known to suppress the growth of NSCLC [14], with JQ1 also having an effect on Kras‐mutant NSCLC [15]. Given the roles played by BET family, variants of BET family genes could potentially influence the survival outcome of cancer patients. Therefore, in the present study, we investigated the effects of rSNPs of BET family genes on the survival outcome of patients with surgically resected NSCLC.

## 2 Materials and methods

### 2.1 Study population

This study was conducted in two stages. In the discovery cohort, we enrolled 349 NSCLC patients who underwent curative surgical resection at the Kyungpook National University Hospital between September 1998 and December 2007. In the validation cohort, we included 424 patients who underwent curative surgery for NSCLC at the Seoul National University Bundang Hospital between September 2005 and March 2012. All of these patients were Korean. Written consent and clinical samples were obtained prior to surgery, and the study was approved by the institutional review boards of both hospitals. Research was performed in accordance with the Declaration of Helsinki of the World Medical Association.

### 2.2 Polymorphism selection and genotyping

To collect polymorphisms, we used the rSNPBase database (http://rsnp.psych.ac.cn/result), which provides a curated human SNP resource with which to analyze the regulatory functions of all SNPs in the human genome and contains references to the regulatory elements with supporting experimental evidence [16]. We retrieved polymorphisms targeting the *BRD2*, *BRD3*, and *BRD4* genes via the “List search” module in rSNPBase. The gene for bromodomain testis‐specific protein was excluded from this study because it is a testis‐specific chromatin protein. In total, 5669, 2025, and 2624 polymorphisms of *BRD2*, *BRD3*, and *BRD4*, respectively, were selected. We excluded SNPs with minor allele frequencies < 0.1 in HapMap JPT and those in strong linkage disequilibrium (LD) (*r* ^2^ > 0.8) using the TagSNP utility for LD‐tag SNP selection in the SNPinfo web server (https://snpinfo.niehs.nih.gov/). Ultimately, 77 SNPs (59 in *BRD2*, 7 in *BRD3*, and 11 in *BRD4*) were selected. These polymorphisms were genotyped using a Sequenom MassARRAY iPLEX assay (Sequenom, San Diego, CA, USA) according to the manufacturer's instructions. *BRD3* rs2427964C>T, which was in strong LD with *BRD3* rs2506711C>T, was genotyped using a TaqMan assay (Thermo Fisher Scientific, Foster City, CA, USA).

### 2.3 Promoter‐luciferase constructs and luciferase assay

To investigate whether the rs2427964C>T variant modulates the promoter activity of the gene, we conducted a luciferase assay. A791‐bp fragment (from −390 to +400 based on rs2427964) that included rs2427964C>T was synthesized by PCR using genomic DNA from a donor carrying a heterozygote. The forward primer with the *KpnI* restriction site (5ʹ‐GGGGTACCGGTTCAGACCTTCCTTAGACC‐3′) and the reverse primer with the *XhoI* restriction site (5′‐CCGCTCGAGAGTTCCCGTTTTGAAATCAGCC‐3′) were used. The PCR products were cloned into the *KpnI*/*XhoI* site of the pGL3‐basic vector (Promega, Madison, WA, USA), which resulted in pGL3‐Basic‐BRD3 constructs containing either rs2427964 C or T alleles. The correct sequences of all the clones were verified by genome sequencing. The H1299 and A549 lung cancer cell lines were transfected with the pRL‐SV40 vector (Promega) and pGL3‐basic vector using Effectene (Qiagen, Hilden, Germany). The cells were harvested 48 h after transfection, and then, lysates were prepared using the Dual‐Luciferase Reporter Assay System (Promega). Luciferase activity was measured using a Synergy HTX Multi‐Mode Microplate Reader (BioTek Instruments, Winooski, VT, USA), and the results were normalized to pRL‐SV40 *Renilla* luciferase activity.

### 2.4 Chromatin immunoprecipitation with quantitative PCR

Chromatin immunoprecipitation (ChIP) assays were performed using the Pierce Magnetic ChIP Kit (Thermo Fisher Scientific) according to the manufacturer’s instructions. Cell lysates from H1299 and A549 were sonicated to reach an average length of 300 bp using Vibra‐Cell Ultrasonic Liquid Processors (Sonics & Materials, Danbury, CT, USA). The DNA–protein complex was then reacted with SIN3A and ETS1 antibodies (Abcam, Cambridge, UK). ChIP DNA was analyzed by quantitative PCR (qPCR) using QuantiFast SYBR Green PCR Master Mix (Qiagen) in a LightCycler 480 (Roche Life Science, Penzberg, Germany). The following primers were used: *BRD3* forward, 5′‐ CCCAGTATCTATTTCTGAGCAG‐3′; *BRD3* reverse, 5′‐ TTCCTCCGTTCGACCCAATCCT‐3′.

### 2.5 Electrophoretic mobility shift assay and supershift assay

An electrophoretic mobility shift assay was performed using the LightShift Chemiluminescent EMSA Kit (Thermo Fisher Scientific) according to the manufacturer's instructions. Nuclear extracts were prepared from H1299 and A549 cells using Pierce NE‐PER Nuclear and Cytoplasmic Extraction Reagent Kit (Thermo Fisher Scientific). Complementary 31‐bp oligonucleotides (based on rs2427964 from −15 to +15) were synthesized: 5′‐CTGAGCAGAAAGCTT**C**CAACCAAAATTAAAG‐ 3′ and 5′‐CTGAGCAGAAAGCTT**T**CAACCAAAATTAAAG‐3′ (the polymorphic alleles are indicated by bold letters). The DNA–protein complex bands were quantified by densitometry analysis using image‐studio 5.2 software (LI‐COR, Lincoln, NE, USA). A supershift assay was performed using 10 μg of nuclear extract incubated with SIN3A and ETS1 antibodies before the labeled probe step.

### 2.6 The Cancer Genome Atlas data analysis

mRNA sequencing data on gene expression were obtained from The Cancer Genome Atlas (TCGA) through the Broad Institute's fire browse data portal for TCGA squamous cell carcinoma and adenocarcinoma cohorts. In total, 51 lung squamous cell carcinoma and 58 lung adenocarcinoma tumor‐paired normal tissue samples were included in our analysis.

### 2.7 Protein extraction and western blotting

H1299 and A549 cell lines were washed twice with prechilled phosphate‐buffered saline (PBS) and then the appropriate amount of mammalian protein extraction reagent (Thermo Fisher Scientific) was added to the cells. Protein concentrations were determined with a Pierce BCA Protein Assay Kit (Thermo Fisher Scientific). Total protein lysates were separated by SDS/PAGE on 10% SDS/acrylamide gels. Subsequently, they were transferred to polyvinylidene difluoride membranes (Merck, Darmstadt, Germany) and then incubated in blocking buffer. BRD3 (Abcam) was used as a primary antibody, whereas *β*‐actin (primary antibody; Santa Cruz Biotech, Santa Cruz, CA, USA) was used as an internal control. Immunoreactive proteins were visualized using Immobilon Western Chemiluminescent HRP Substrate (Merck).

### 2.8 BRD3 small interfering RNAs and transfection

BRD3 knockdown in human lung cancer cells was achieved by the transfection of BRD3 small interfering RNA (siRNA), denoted siBRD3, which was synthesized by Bioneer (Daejeon, Korea). The siRNA sequence targeting *BRD3* corresponded to the coding regions (5′‐CUGAUGUCCGGCUGAUGUU‐3′; antisense 5′‐AACAUCAGCCGGACAUCAG‐3′) of the *BRD3* gene. A nonspecific siRNA oligonucleotide from Bioneer was used as a negative control (siCtrl), whereas untreated cells or cells treated only with transfection reagents were used as controls (Mock). Cells in the exponential phase of growth were plated. After 24 h, cells were transfected with 100 nm of siRNA using Effectene transfection reagent (Qiagen) according to the manufacturer's protocol.

### 2.9 Cell proliferation assay

*In vitro* cell proliferation was tested using a 3‐(4,5‐dimethylthiazol‐2‐yl)‐2,‐5‐diphenyltetrazolium bromide (MTT) assay. First, cells were seeded at a density of 4 × 10^3^ cells per well in 96‐well culture plates. After 24 h, the medium was removed and the cells were transfected with siBRD (Bioneer) using Effectene (Qiagen). Cell viability was then determined at different time points using a Multiskan FC Microplate Photometer (Thermo Fisher Scientific). The cell viability in each well was measured in terms of optical density at a wavelength of 570 nm.

### 2.10 Transwell invasion assay

Cell invasion assays were performed using BioCoat Matrigel invasion chambers (24‐well; 8‐μm pore size; Corning, NY, USA). Before the experiment, Matrigel‐coated Transwell inserts were rehydrated in 500 μL of serum‐free RPMI‐1640 medium and incubated at 37 °C for 2 h. First, H1299 and A549 cells were trypsinized and washed with PBS; subsequently, 2.5 × 10^4^ cells were suspended in 500 μL of RPMI‐1640 without fetal bovine serum and the suspension was added to the upper chamber, whereas 750 μL of RPMI‐1640 containing 10% fetal bovine serum was added to the lower chamber. The cells were allowed to invade for 12 h, after which they were fixed with 3.7% paraformaldehyde for 20 min, washed with PBS, and then stained with 0.05% crystal violet in 20% methanol for 30 min. The Transwell inserts were washed with PBS and the noninvasive cells in the upper chambers were removed with cotton swabs. Representative images were acquired using an inverted microscope (Nikon, Tokyo, Japan), and the percentage of stained cells was measured using an image analyzer (image‐studio 5.2).

### 2.11 Wound‐healing assay

H1299 and A549 cells were seeded in separate 6‐well plates and allowed to grow until confluence. The confluent monolayers of these cells were gently scratched using sterile 20‐μL pipette tips before being rinsed with PBS to remove cellular debris. Cells were then treated with the previously indicated concentrations of siRNA, after which the 6‐well plates were incubated at 37 °C in 5% CO_2_ for 0, 24, and 48 h. For each time point, the scratched area was then observed and photographed under an inverted microscope, and the distance of the scratch closure was examined. Wound closure was measured using the MRI wound‐healing tool plugin for imagej (https://github.com/MontpellierRessourcesImagerie/imagej_macros_and_scripts/wiki/Wound‐Healing‐Tool). To generate a wound closure percentage, measurements were normalized to the initial scratch area (0 h measurement = 100%) for each replicate.

### 2.12 Cell apoptosis analysis with an annexin V assay

H1299 and A549 cells transfected with siBRD3 or scrambled siRNA were gently dissociated with trypsin/EDTA and stained for annexin V and propidium iodide using the Annexin V‐FITC Apoptosis Detection Kit (Abcam) according to manufacturer's instructions. The stained cells were immediately analyzed with a BD FACS Calibur system (BD Biosciences, Heidelberg, Germany).

### 2.13 Statistical analyses

The OS data were analyzed with the Kaplan–Meier method. The OS was calculated from the day of surgery to the day of death or the last follow‐up. Adjusted hazard ratio (aHR) and 95% confidence intervals (CIs) were calculated for multivariate statistical models (Cox proportional hazards models) with adjustments for age, gender, smoking status, pathologic stage, and adjuvant therapy. *P* < 0.05 was considered statistically significant. All analyses were performed using Statistical Analysis System for Windows, version 9.4 (SAS Institute, Cary, NC, USA).

## 3 Results

### 3.1 Associations between polymorphisms and survival outcomes

The characteristics of the patients and the associations with OS are shown in Table S1. Pathologic stage had a statistically significant effect on OS in both cohort groups. Younger age was associated with better OS in the discovery cohort; female, never smoking, and adenocarcinoma were associated with better OS in the validation cohort (Table S1).

Of the 77 SNPs genotyped, 59 were analyzed after excluding 18 SNPs with genotype failure or deviations from Hardy–Weinberg equilibrium (Table S2). In the discovery cohort, six SNPs were significantly associated with OS (*P* < 0.05) (Table S3). Among these six SNPs, only *BRD3* rs2506711C>T was replicated with the same direction as the discovery cohort in the validation cohort. *BRD3* rs2506711C>T was significantly associated with worse OS in the discovery cohort, validation cohort, and combined analysis (under a recessive model; aHR = 1.79, 95% CIs = 1.17–2.74, *P* = 0.01; aHR = 1.97, 95% CIs = 1.18–3.28, *P* = 0.01; and aHR = 1.91, 95% CIs = 1.38–2.65, *P* = 9 × 10^−5^, respectively; Table 1).

**Table 1:** OS according to genotypes of the rs2506711C>T and rs2427964C>T in the discovery, validation, and combined cohorts. MAF, minor allele frequency; no, number.

Polymorphism target gene	Genotypes	Discovery cohort				Validation cohort				Combined analysis			
		no	MAF	aHR (95% CIs)	*P* ^a^	no	MAF	aHR (95% CIs)	*P* ^a^	no	MAF	aHR (95% CIs)	*P* ^a^
rs2506711^b^ *BRD3*	CC	113	0.44	1.00		125	0.44	1.00		238	0.44	1.00	
	CT	163		1.12 (0.70–1.78)	0.64	222		1.00 (0.59–1.69)	0.99	385		1.04 (0.74–1.46)	0.83
	TT	72		1.92 (1.15–3.21)	0.01	73		1.97 (1.07–3.60)	0.03	145		1.96 (1.33–2.89)	1 × 10^−3^
	Dominant	235		1.33 (0.87–2.05)	0.19	295		1.21 (0.74–1.98)	0.45	530		1.26 (0.91–1.73)	0.16
	Recessive	276		1.79 (1.17–2.74)	0.01	347		1.97 (1.18–3.28)	0.01	623		1.91 (1.38–2.65)	9 × 10^−5^
	Codominant			1.39 (1.06–1.82)	0.02			1.39 (1.00–1.93)	0.05			1.39 (1.13–1.72)	2 × 10^−3^
rs2427964^b^ *BRD3*	CC	115	0.44	1.00		127	0.44	1.00	0.97	242	0.44	1.00	
	CT	159		1.21 (0.76–1.93)	0.43	220		1.01 (0.60–1.71)	0.97	379		1.08 (0.76–1.53)	0.66
	TT	74		1.91 (1.13–3.20)	0.02	73		1.98 (1.08–3.63)	0.03	147		2.01 (1.36–2.97)	4 × 10^−4^
	Dominant	233		1.40 (0.91–2.17)	0.13	293		1.22 (0.75–2.00)	0.42	526		1.31 (0.95–1.8)	0.10
	Recessive	274		1.70 (1.11–2.61)	0.02	347		1.97 (1.18–3.28)	0.01	621		1.92 (1.39–2.65)	8 × 10^−5^
	Codominant			1.38 (1.06–1.81)	0.02			1.39 (1.01–1.94)	0.05			1.42 (1.15–1.74)	1 × 10^−3^

^a^ *P*‐values were calculated using multivariate Cox proportional hazard models, adjusted for age, gender, smoking status, tumor histology, pathologic stage, and adjuvant therapy.

^b^ rs2506711 and rs2427964 are in strong LD (D′ = 0.99 and *r* ^2^ = 0.98).

**Fig. 1:**
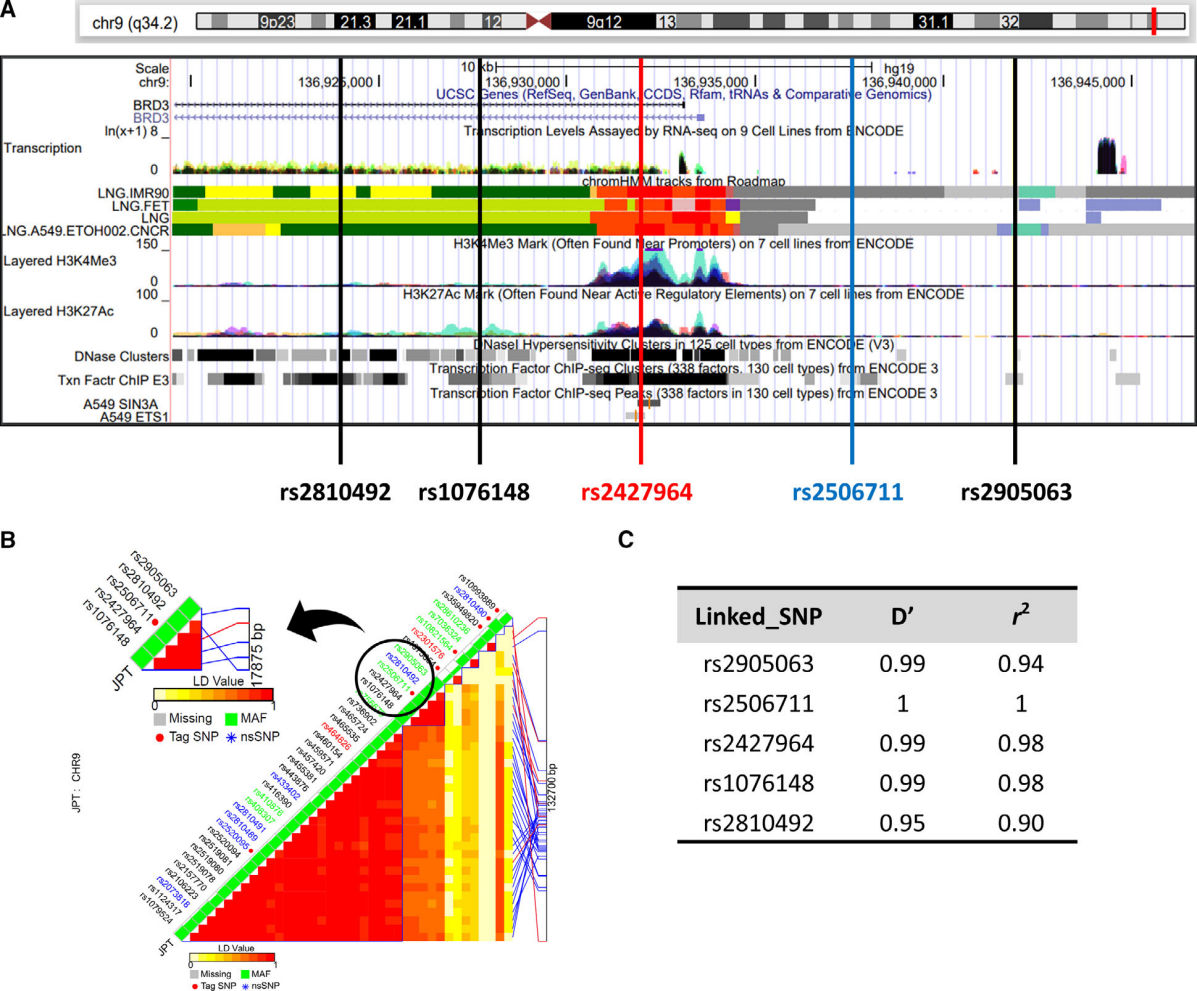
Bioinformatics annotation of BRD3 promoter region using UCSC genome browser and five polymorphisms in LD. (A) UCSC genome browser view of chromosome 9q34.2 with the transcription factor ChIP‐seq, DNase 1 hypersensitivity, histone modifications from the ENCODE project [24], and the chromatin state segmentation tracks from the RoadMap Epigenome project. Definitions of track colors are listed in Fig. S3. The chromosomal positions of rs2506711 and four LD single nucleotide polymorphisms (SNPs) are shown as vertical lines. The histone modification tracks show the levels of enrichment of the histone mark [H3K4 me3 mark (TSS) and H3K27Ac mark (TSS and Enhancer)] across the genome as determined by a ChIP‐seq assay using the seven cell lines of the ENCODE project. The next track shows DNase I hypersensitivity clusters. The last track represents transcription factor ChIP‐seq clusters (338 factors from 130 cell types) from ENCODE 3. This track shows regions of transcription factor binding derived from a large collection of ChIP‐seq experiments performed in the ENCODE project. A gray box encloses each peak cluster of transcription factor occupancy; the darkness of the box is proportional to the maximum signal strength observed in any cell type contributing to the cluster. Transcription factors SIN3A and ETS1 bind to rs2427964. (B) *BRD3* tagging SNP plot generated by SNPinfo Web Server: LD‐tag SNP selection web‐based software using JPT (Japanese in Tokyo). rs2506111 was the tagged SNP and rs2427964 was the SNP selected among five SNP showing strong LD. Shading represents the magnitude and significance of pairwise LD, with a red‐to‐white gradient reflecting higher‐to‐lower LD values. (C) Table showing the LD analysis of four SNPs in LD with rs2506711. LD was calculated based on 1,000 genomes JPT data.

**Fig. 2:**
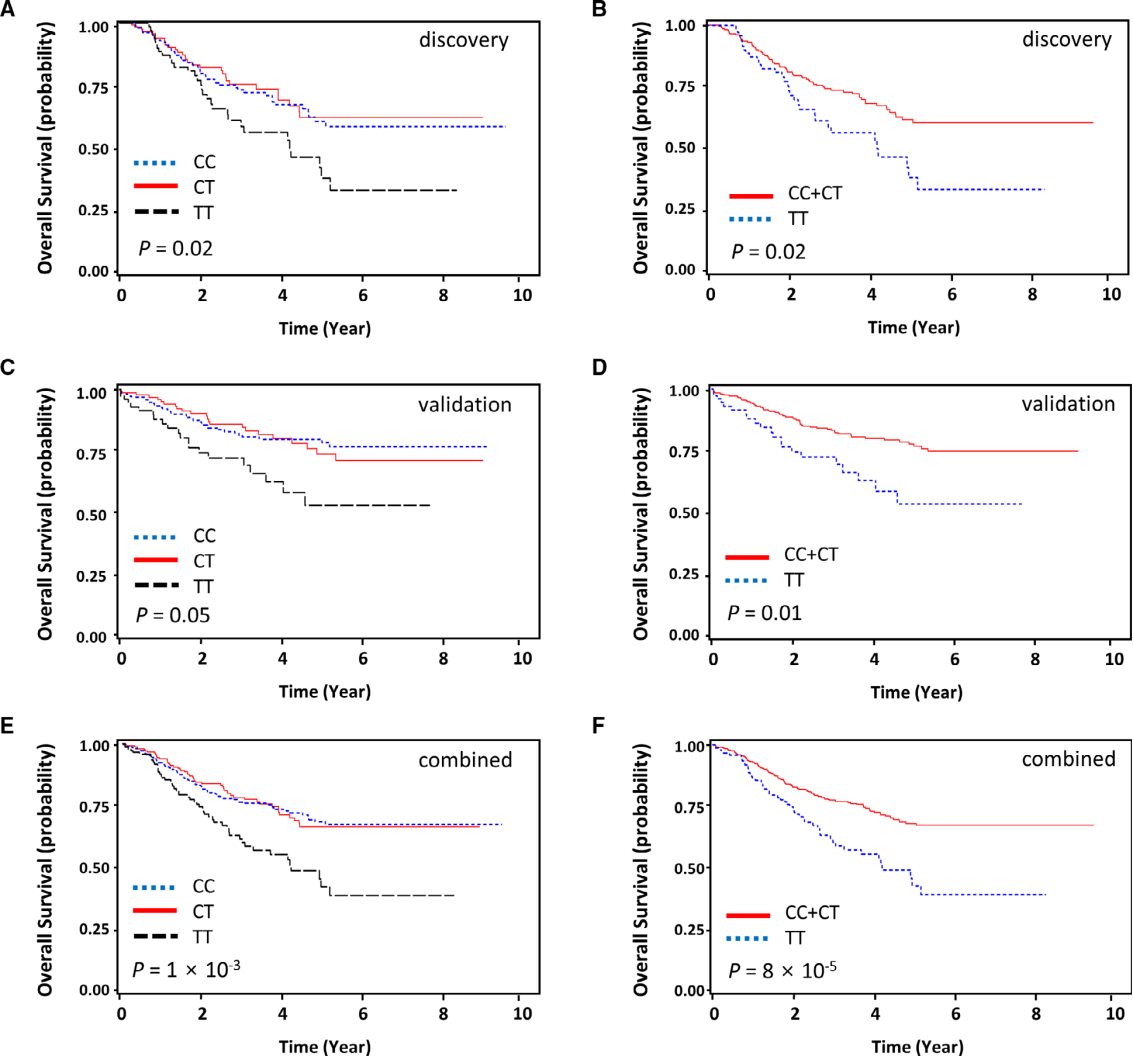
OS curves according to the *BRD3* rs2427964 genotypes. The discovery cohort (A, B), validation cohort (C, D), and combined analysis (E, F). *P* values were calculated using a multivariate Cox proportional hazard model.

According to annotations including histone modification signals, DNase, and transcription factor binding sites in ChIP‐seq data from the ENCODE project, rs2506711C>T is located in heterochromatin and the repressed area (Fig. 1A and S3). Therefore, the effect of rs2506711C>T on OS may be due to other causal SNPs in LD. Although rs2506711C>T is a tagged variant, four different SNPs are in a strong LD relationship (*D*′ and *r* ^2^ ≥ 0.9) with rs2506711 (Fig. 1B,C). The functional annotations of five SNPs in LD are shown in Fig. 1 and Table S4. Among these five LD SNPs, *BRD3* rs2427964 is located in a potential regulatory region, including various transcription factor binding sites (combined with multiple cell lines), with high chromatin accessibility as measured by DNaseI hypersensitivity, and a strong signal for an active histone marker (H3K4Me3 and H3K27Ac). *BRD3* rs2427964 has strong LD with rs2506711 (D′ = 0.99 and *r* ^2^ = 0.98), is located in the transcription factor binding region, and is predicted to act as a promoter (Table S4). *BRD3* rs2427964C>T was also associated with worse OS in the discovery cohort, validation cohort, and combined analysis (under a recessive model; aHR = 1.70, 95% CIs= 1.11–2.61, *P* = 0.02; aHR = 1.97, 95% CIs = 1.18–3.28, *P* = 0.01; and aHR = 1.92, 95% CIs = 1.39–2.65, *P* = 8 × 10^−5^, respectively; Table 1 and Fig. 2).

In stratified analysis, the effect of *BRD3* rs2427964 on survival outcomes differed according to histological cell type (Table S5). Significant associations between *BRD3* rs2427964 and worse OS were found in adenocarcinoma (aHR = 2.78, 95% CIs = 1.77–4.35, *P* = 8× 10^−6^) but not in squamous cell carcinoma (aHR= 1.35, 95% CIs = 0.81–2.25, *P* = 0.25; *P* < 0.05 for the homogeneity test; Table S5).

### 3.2 Effect of BRD3 rs2427964 on promoter and transcription activity

**Fig. 3:**
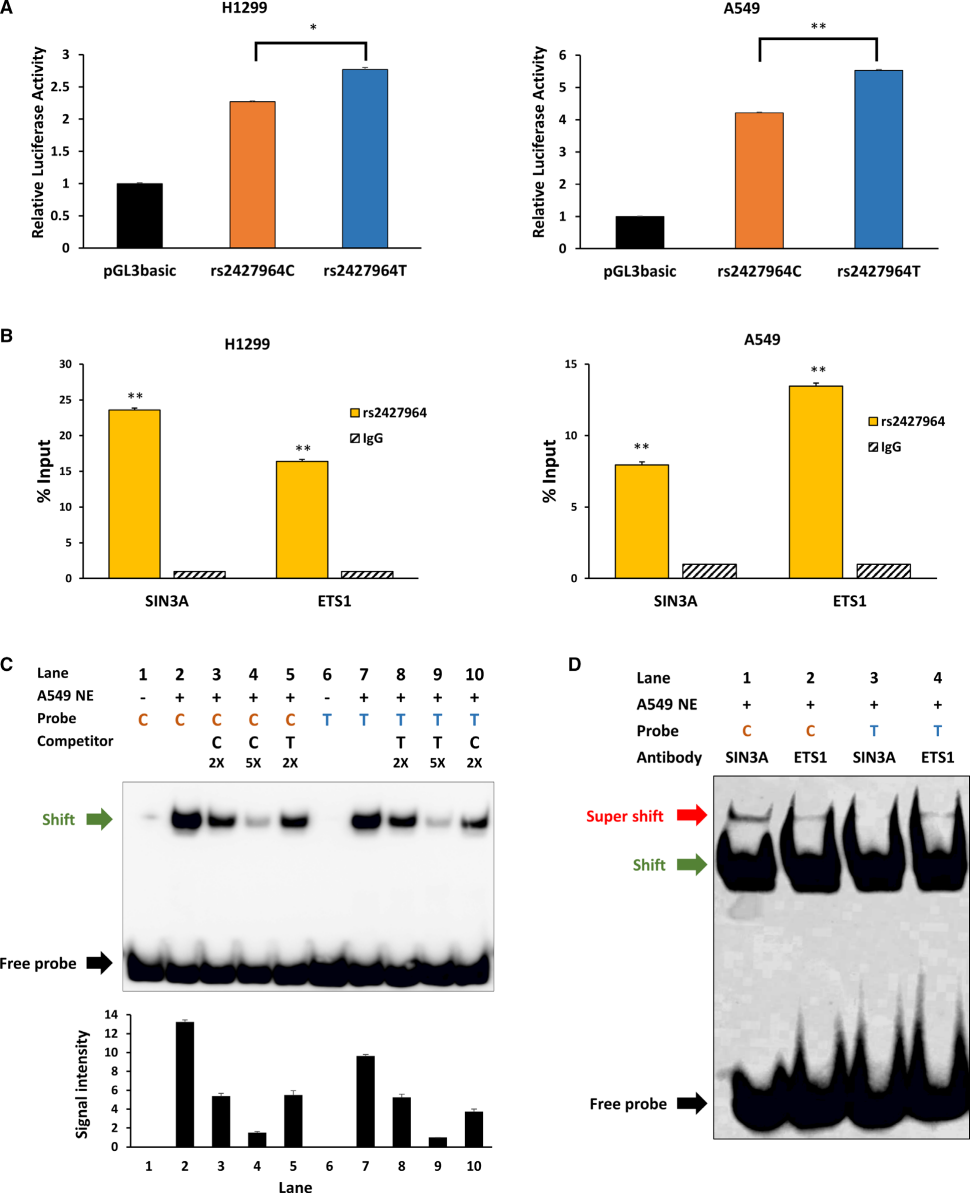
Effect of BRD3 rs2427964C>T on promoter and transcription activity. (A) The effect of rs2427964C>T genotypes on the luciferase activity of the *BRD3* promoter. The luciferase activity of rs2427964 C and T alleles was measured using a dual‐luciferase reporter assay system in H1299 and A549 cell lines. Data are presented as mean ± SEM (*n* = 8). *P* values were derived from a *t*‐test; *P < 0.05, ***P < 0.005. (B) Chromatin immunoprecipitation quantitative PCR analysis of sequences containing the SNP rs2427964 in H1299 and A549 cell lines. Quantitative PCR was used to amplify chromatin derived from immunoprecipitations with rabbit IgG, anti‐SIN3A antibody, and anti‐ETS1 antibody. Data are presented as mean ± SEM (*n* = 4). **P < 0.005 calculated by Student's *t*‐test. (C) Electrophoretic mobility shift assay with rs2427964 alleles. Nuclear extract protein from the A549 cell line was incubated with biotin‐labeled C or T allele probes for the rs2427964‐containing region. Competition assays were performed using unlabeled rs2427964‐C or rs2427964‐T oligonucleotides. Each binding reaction contained 5 μg of nuclear extract, except in lanes 1 and 6, and labeled probes of the rs2427964‐C (lanes 1–5) or rs2427964‐T (lanes 6–10) oligonucleotides. Shift band signal intensities were analyzed by Image‐Studio lite software (LI‐COR Biosciences, Lincoln, NE, USA). Data represent the mean ± SEM (*n* = 3). (D) A supershift assay was performed using SIN3A and ETS1 antibodies. Each binding reaction contained 10 μg of nuclear extract and labeled probes of the rs2427964‐C (lanes 1–2) or rs2427964‐T (lanes 3–4) oligonucleotides. Representative data from 3 experiments are shown.

The variant rs2427964C>T is estimated to be a promoter region of BRD3; thus, the effect of rs2427964C>T on BRD3 promoter activity was assessed *in vitro* using a luciferase assay. Promoter activity was significantly increased with the rs2427964 T allele relative to with the rs2427964 C allele in both H1299 and A549 cell lines (Fig. 3A).

Using publicly available ChIP‐seq data from the ENCODE database, we searched several transcription factors that were identified to bind over rs2427964. According to the transcription factor ChIP‐seq data set of A549 cells, rs2427964 is located in SIN3A and ETS1 binding regions (Fig. 1A). In this study, we confirmed that the rs2427964‐containing region was in SIN3A and ETS1 binding regions not only in the A549 cell line but also in the H1299 cell line. The input ratio using ChiP‐qPCR was higher in the rs2427964‐containing region compared with that in the control (IgG) in both H1299 and A549 cell lines (Fig. 3B).

An electrophoretic mobility shift assay was performed to determine differences in binding to transcription factors according to the rs2427964 alleles. As shown in Fig. 3C, the rs2427964 C allele (lane 2) bound more transcription factors than were bound with the rs2427564 T allele (lane 7). The density of shift of a biotin‐labeled T allele probe was reduced with a competitor C allele (lane 10) compare with the effect observed with a competitor T allele (lane 8). In contrast, the density of shift of a biotin‐labeled C allele probe was increased with a competitor T allele (lane 5) relative to the effect with a competitor C allele (lane 3). The transcription factors SIN3A and ETS1 are known to bind to rs2427964. To assess whether the degree of binding to SIN3A and ETS1 differed depending on the rs2427964 C or T alleles, we conducted a supershift assay. As shown in Fig. 3D, the EST1 antibody bound to both C and T alleles but the SIN3A antibody selectively bound to the C allele. Since SIN3A is known as a transcription repressor [17], the binding between the *BRD3* rs2427964 C allele and SIN3A is expected to reduce BRD3 expression. To determine whether SIN3A is functional in conjunction with BRD3, we conducted an experiment on siRNA‐mediated knockdown of the *SIN3A* gene in H1299 and A549 lung cancer cell lines. We confirmed via RT‐PCR and western blot that the expression of BRD3 mRNA and protein increased when SIN3A expression was reduced using siRNA (Fig. S1A,B).

### 3.3 BRD3 expression in cancer cell lines and effects on survival

**Fig. 4:**
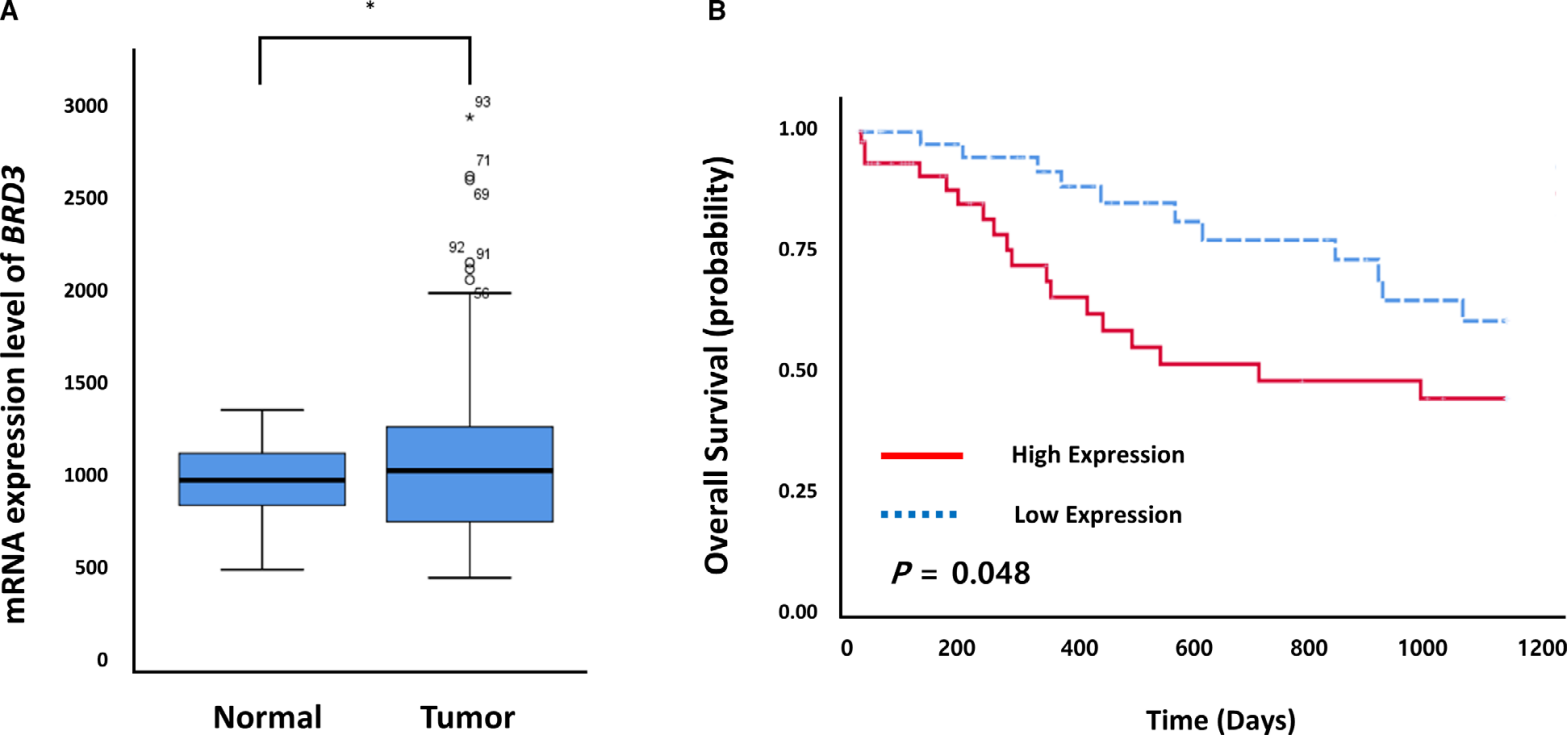
BRD3 expression and survival data obtained from The Cancer Genome Atlas (TCGA) database. (A) The mRNA expression level of BRD3 in tumor and nonmalignant lung tissues as determined via TCGA database. The mRNA expression level was obtained from 109 lung cancer patients (51 with lung squamous cell carcinoma and 58 with lung adenocarcinoma). *P < 0.05. (B) Kaplan–Meier plots of OS according to BRD3 mRNA expression determined via TCGA database. High‐ and low‐expression groups were defined by the median BDR3 mRNA expression value of the tumor tissues.

To explore the potential role of *BRD3*, we first examined BRD3 mRNA expression in cancer cell lines. Cancer cell encyclopedia data showed that BRD3 mRNA expression levels were elevated in various human cancer cell lines including lung cancer (Fig. S2A). Thus, we measured BRD3 mRNA expression in various lung cancer cell lines using RT‐PCR and found that expression was elevated in several cell lines (Fig. S2B). In addition, TCGA data showed that BRD3 mRNA expression was significantly higher in lung cancers than the expression in adjacent normal tissues (Fig. 4A). Moreover, OS was worse in patients showing high BRD3 expression compared with OS in those showing low BRD3 expression (*P* = 0.048, Fig. 4B).

### 3.4 Effects of BRD3 silencing on cancer cells

**Fig. 5:**
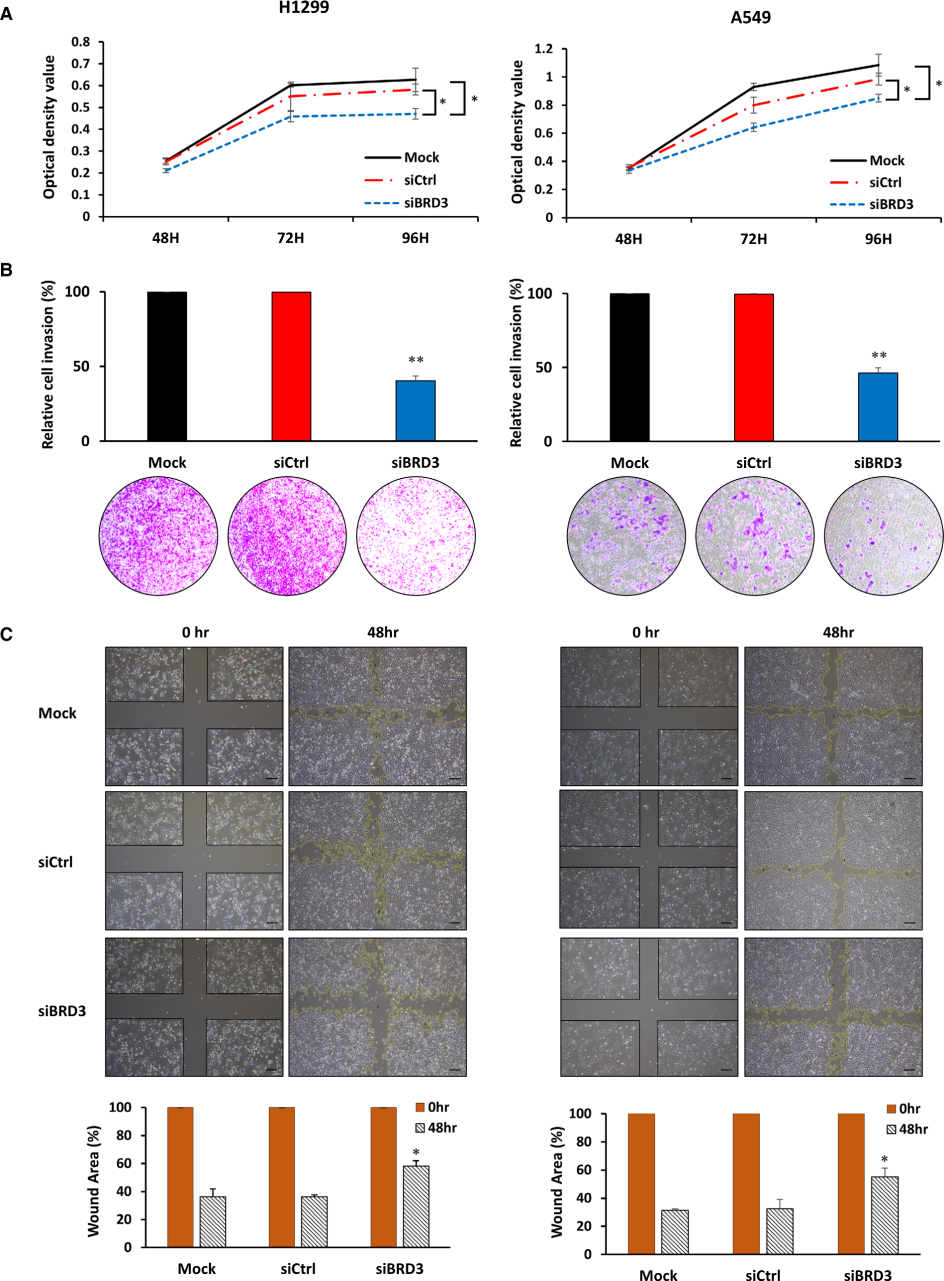
Effects of BRD3 silencing on H1299 and A549 cells. (A) Cell viability as evaluated by an MTT assay (*n* = 3); siRNA was applied 24 h after cell seeding. (B) After transfection with Mock, siRNA control (siCtrl), and siBRD3, the cell invasion ability of H1299 and A549 cells was assessed using a Transwell invasion assay. The number of Mock cells was set to 100%. Representative images from 3 experiments are shown below the graph. (C) A wound‐healing assay (*n* = 4) was performed to detect the migration of cells. Upper: representative photographs of controls and siBRD3‐treated H1299 and A549 cells in the wound‐healing assay. Scale bar: 100um. Lower: percentage of wound area as determined in imagej by the rate of cells moving toward the scratched area over a given period. *P* values are derived from comparisons with the Mock control group. Data are presented as mean ± SEM and two‐tailed Student's *t*‐test was used to calculate the statistical significance: *P < 0.01, **P < 0.001.

To evaluate the knockdown efficacy of BRD3, we used three commercial siRNAs. After transfection with siBRD3, the level of BRD3 mRNA expression decreased significantly compared with levels in the control (Fig. S1C). Western blots showed that BRD3 expression was significantly reduced in H1299 and A549 cells after transfection with siBRD3 (Fig. S1D). Since BRD3 is highly expressed in lung cancer, we tested whether BRD3 knockdown affected lung cancer cell proliferation. As shown in Fig. 5A, when siBRD3 was transfected, cell proliferation was significantly reduced in both H1299 and A549 cell lines.

The effect of BRD3 knockdown on cell invasion ability was investigated using a Transwell invasion assay after transfection with siBRD3. H1299 and A549 cell invasion was significantly repressed in the siBRD3 group compared with cell invasion in the Mock and siCtrl groups (Fig. 5B). A wound‐healing assay also showed that cell migration was reduced after transfection with siBRD3 for 48 h (Fig. 5C).

**Fig. 6:**
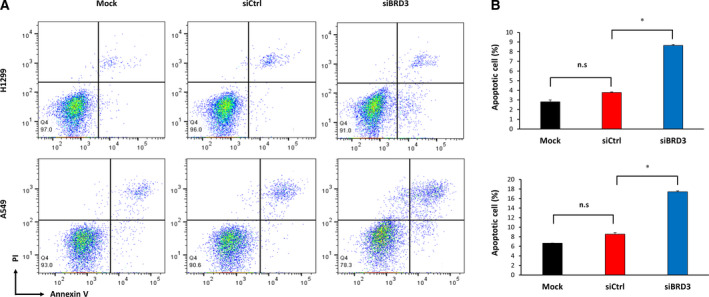
Effect of *BRD3* knockdown‐induced apoptosis. (A) Flow cytometry results with Annexin V‐FITC/PI staining. (B) The ratio of apoptosis among different experimental groups. Apoptosis ratio was calculated using the early apoptosis percentage plus the late apoptosis percentage. Columns: means of four independent experiments; bars: standard deviation. *P* values are derived from comparisons with the Mock control group by *t*‐test: *P < 0.01 and n.s. not significant.

Annexin V‐FITC double staining and flow cytometry analysis were performed to determine whether BRD3 silencing had an effect on cell apoptosis. In BRD3‐silenced H1299 and A549 cells, the percentage of apoptotic cells increased in comparison with the percentage in the control group (Fig. 6A,B).

## 4 Discussion

As epigenetic readers, BET proteins are critical regulators of transcription. Bromodomain proteins bind to acetylated histone lysine residues and mediate downstream functions such as histone acetylation, chromatin remodeling, and transcriptional regulation [18]. BET proteins are also involved in cancer pathogenesis or progression through promotion of aberrant oncogene expression [9]. For example, BRD4 regulates breast cancer dissemination through Jagged1/Notch1 signaling [19]. The proto‐oncogene MYC is also associated with BET proteins; BET inhibitor downregulates MYC transcription leading to therapeutic effects on multiple myeloma and medulloblastoma [20, 21].

In the present study, *BRD3* rs2427964C>T was associated with worse OS in both the discovery and validation cohorts. BRD3 plays a role in the regulation of transcription, probably through chromatin remodeling and interactions with transcription factors [22]. Although the *BRD3*‐*NUT* fusion gene has been reported in NUT midline carcinoma, the role of BRD3 in cancers is not well known. According to TCGA data, BRD3 expression was higher in lung cancer tissues than it was in tumor‐paired normal tissue. Moreover, OS was worse in patients with high BDR3 expression. Following knockdown of BRD3 with BRD3‐specific siRNA, we found that the growth of lung cancer cells (H1299 and A549) decreased over time. Cell invasion and migration were also inhibited after transfection with siBRD3. Taken together, these results suggest that BRD3 plays important roles in cancer cell growth and migration. Furthermore, we found that BRD3 silencing increased the apoptosis rate in lung cancer cells compared with the rate observed in the control. Therefore, in a similar manner to BRD4‐targeting inhibitors suppressing NSCLC growth [14], drugs targeting BRD3 could potentially treat lung cancer.

Our genotype analysis showed that patients with the rs2427964TT genotype had worse survival outcomes than those with the rs2427964CC/CT genotype. This result is biologically plausible because (a) TCGA data showed that patients with high BRD3 expression had worse prognosis than those with low BRD3 expression and (b) BRD3 silencing decreased the proliferation and migration of cancer cells. Thus, a lack of BRD3 expression or at least low expression seems to improve prognosis. We also showed that the rs2427964 T allele had higher promoter activity than that of the rs2427964 C allele. There is a possibility, according to our findings, that differences in binding to the transcriptional repressor SIN3A related to the rs2427964 allele could be associated with differences in BRD3 expression. In summary, *BRD3* rs2427964C>T may increase BRD3 expression through an increase in promoter activity; this likely leads to poor prognosis for lung cancer patients. In additional analysis, BRD3 expression showed a tendency to increase in the rs2427964 T allele in 124 lung cancer tissues, but this trend was not statistically significant (data not shown).

Unlike BRD2 or BRD4, the functional role of BRD3 in carcinogenesis is not currently clear. A recent study showed that BRD2 and BRD3 are colocalized with the architectural/insulator protein CCCTC‐binding factor (CTCF), which serves to increase the boundary function of CTCF [23]. The same study suggested that BRD2 or BRD3 defects could lead to improper coregulation of genes that normally remain isolated as well as increasing ectopic interactions across domain boundaries [23]. BET inhibitors preferentially suppress the transcription of cancer‐promoting genes and show clinical effects in several human cancers [10, 13, 14]. As mentioned earlier, BRD3 may affect cancer prognosis by controlling the expression of oncogenes, such as MYC, in a similar manner to BRD4. Nevertheless, further research is required to determine whether the BRD3 abnormalities themselves affect cancer prognosis or whether they affect cancer prognosis through the interactions of other epigenetic modulators and/or oncogenes.

## 5 Conclusion

In summary, the *BRD3* rs2427964C>T allele was associated with survival outcomes for surgically resected NSCLC patients. The patients with the rs2427964TT genotype had worse survival outcomes than those with the rs2427964CC or CT genotype. Specifically, the rs2427964 C allele was selectively bound with a transcriptional repressor and showed lower promoter activity. Additionally, BRD3 mRNA expression was higher in lung cancers than the expression in normal tissues and patients with high BRD3 expression had worse OS. We also found that BRD3 silencing decreased the proliferation and migration of cancer cells. These results suggest that worse survival outcome in NSCLC may be related to an increase in BRD3 expression associated with the change of *BRD3* rs2427964C to rs2427964T.

## Conflict of interest

The authors declare no conflict of interest.

### Peer Review

The peer review history for this article is available at https://publons.com/publon/10.1002/1878‐0261.13109.

### Author contributions

JHL., SSY, MJH, and JYP conceived and designed the study; SSY, SHC, YHL, HWS, JHL, SYL, SIC, CHK, SKC, SHJ, and JYP contributed provision of study material or patients; JHL, SSY, MJH, JEC, HGK, SKD, SHC, YHL, and HWS collected the data; JHL, SSY, MJH, JEC, WKL, SYL, SIC, CHK, and JYP analyzed and interpreted the data; and JHL, SSY, MJH, and JYP wrote the manuscript.

## Supporting information


**Fig. S1**. Effects of SIN3A knockdown on BRD3 mRNA and protein expression. (A) RT‐PCR analysis (*n* = 4) of SIN3A mRNA knockdown via 100‐nm siRNA in H1299 and A549 cells, and BRD3 mRNA expression after knockdown of SIN3A. Columns: means of independent experiments; bars: standard deviations. *P* values are derived from comparisons with the Mock control group by *t*‐test: *P < 0.001. (B) BRD3 protein expression, according to western blots, after transfection with siSIN3A; *β*‐actin was used as an internal control. (C) RT‐PCR analysis (*n* = 4) of *BRD3* mRNA knockdown by 100‐nm siRNA in A549 cells. *BRD3* mRNA expression decreased after transfection with three commercial *BRD3* siRNAs (siBRD3) compared with expression in the control. *P* values are derived from comparisons with the Mock control group by *t*‐test: *P < 0.01. (D) The expression of *BRD3* protein after transfection with siBRD3 by western blots; *β*‐actin was used as an internal control.

**Fig. S2**. Expression of BRD3 in cancer cell lines. (A) Box plots showing RNA‐seq data of mRNA expression from the Cancer Cell Line Encyclopedia: dashed lines within a box represent the mean. Cell lines from the same area or system of the body are grouped together; lineages are indicated at the bottom of the graph; the number of cell lines is shown in parenthesis. (B) Expression of BRD3 in various lung cancer cell lines according to RT‐PCR analysis. MRC5 cell line: human lung fetal fibroblast cells.

**Fig. S3**. Definition of track colors of chromHMM tracks from the Roadmap Consortium. This track displays the chromatin state segmentation for the cell types used by the Roadmap Consortium. In total, 15 states (Primary), 18 states (Auxiliary), and 25 states (Imputed) were used to segment the genome; these states were then grouped and colored to highlight the predicted functional elements.

**Table S1**. Univariate analysis for OS by clinicopathologic features in the discovery and validation cohorts.
**Table S2**. Summary of studied polymorphisms.
**Table S3**. Six polymorphisms associated with OS in the discovery cohorts with *P* < 0.05 and their validation.
**Table S4**. Summary of the functional annotation for five polymorphisms within LD.
**Table S5**. Stratified analysis of the effects of rs2427964C>T genotypes under a recessive model on survival outcomes.

## Data Availability

The datasets and materials in this study are available on reasonable request from corresponding authors or first author.
