# Supplementary figures and images for: Epigenetic readers and lung cancer: the rs2427964C>T variant of the bromodomain and extraterminal domain gene BRD3 is associated with poorer survival outcome in NSCLC
Source: Mol Oncol. 2021 Oct 15;16(3):750–63. doi: 10.1002/1878-0261.13109 (PMC8807359; doi:10.1002/1878-0261.13109)

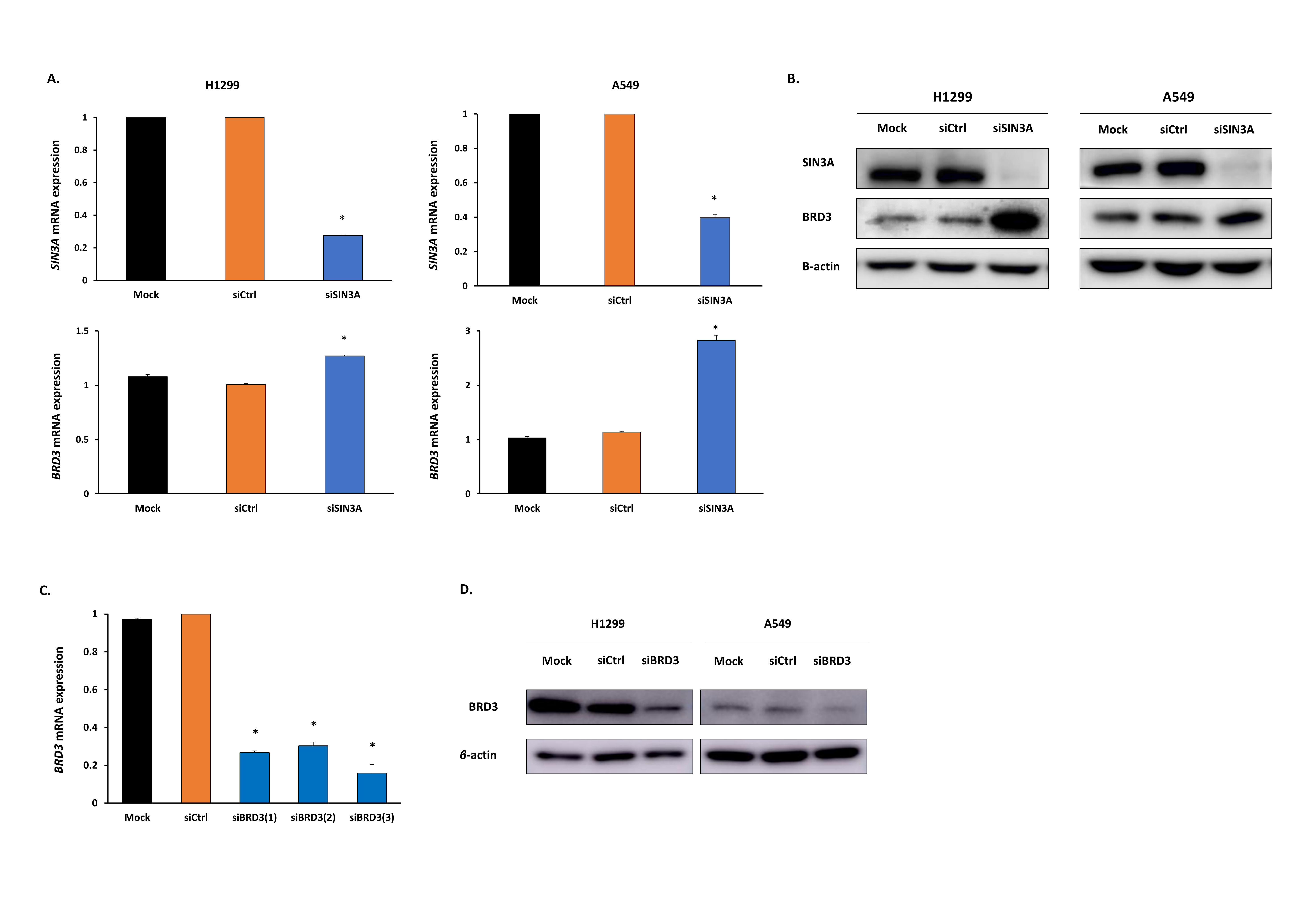

Supplement: Supplementary file 1 — Fig. S1. Effects of SIN3A knockdown on BRD3 mRNA and protein expression. (A) RT‐PCR analysis (n = 4) of SIN3A mRNA knockdown via 100‐nm siRNA in H1299 and A549 cells, and BRD3 mRNA expression after knockdown of SIN3A. Columns: means of independent experiments; bars: standard deviations. P values are derived from comparisons with the Mock control group by t‐test: *P < 0.001. (B) BRD3 protein expression, according to western blots, after transfection with siSIN3A; β‐actin was used as an internal control. (C) RT‐PCR analysis (n = 4) of BRD3 mRNA knockdown by 100‐nm siRNA in A549 cells. BRD3 mRNA expression decreased after transfection with three commercial BRD3 siRNAs (siBRD3) compared with expression in the control. P values are derived from comparisons with the Mock control group by t‐test: *P < 0.01. (D) The expression of BRD3 protein after transfection with siBRD3 by western blots; β‐actin was used as an internal control. [file MOL2-16-750-s003.tiff]

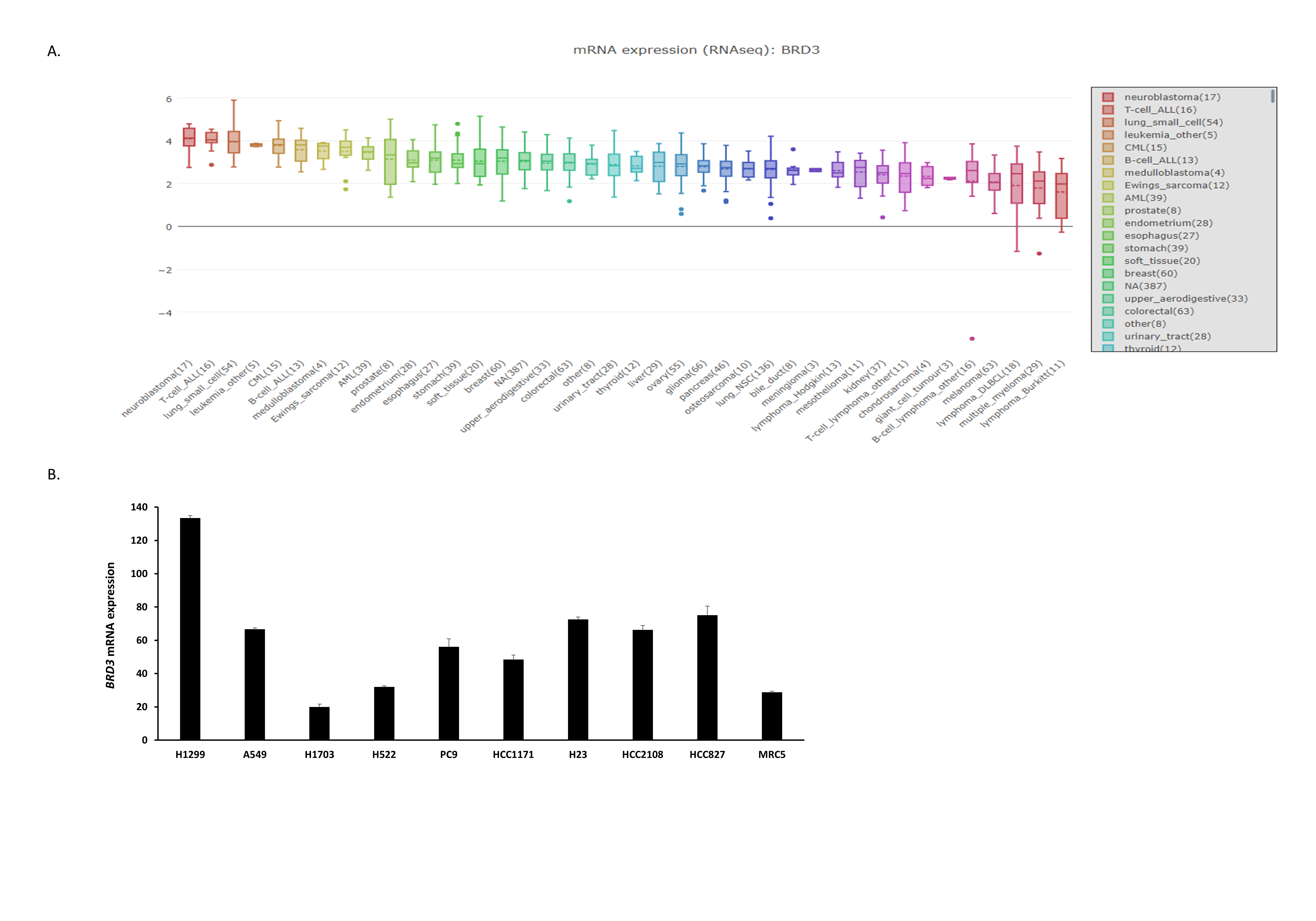

Supplement: Supplementary file 2 — Fig. S2. Expression of BRD3 in cancer cell lines. (A) Box plots showing RNA‐seq data of mRNA expression from the Cancer Cell Line Encyclopedia: dashed lines within a box represent the mean. Cell lines from the same area or system of the body are grouped together; lineages are indicated at the bottom of the graph; the number of cell lines is shown in parenthesis. (B) Expression of BRD3 in various lung cancer cell lines according to RT‐PCR analysis. MRC5 cell line: human lung fetal fibroblast cells. [file MOL2-16-750-s001.TIF]

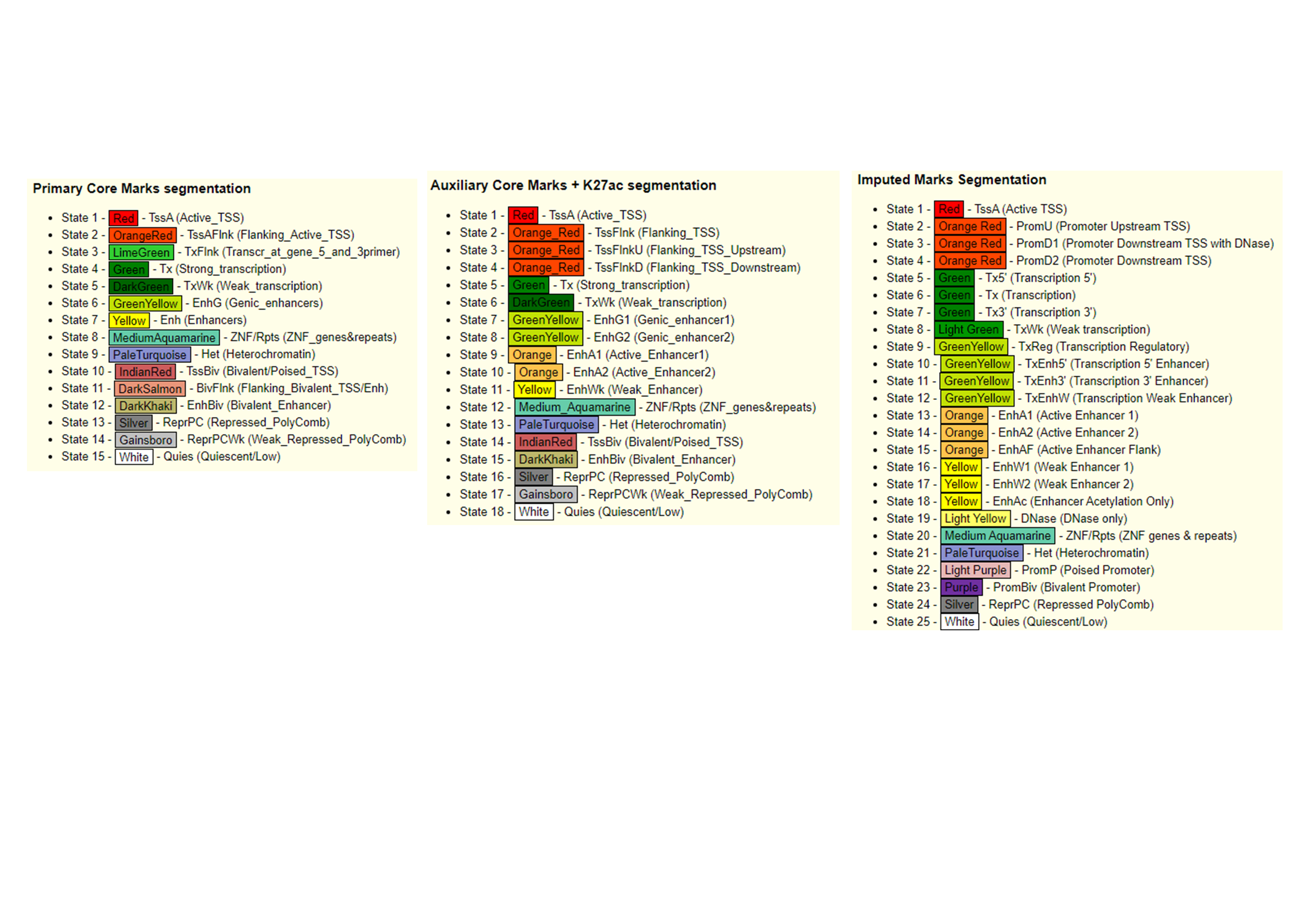

Supplement: Supplementary file 3 — Fig. S3. Definition of track colors of chromHMM tracks from the Roadmap Consortium. This track displays the chromatin state segmentation for the cell types used by the Roadmap Consortium. In total, 15 states (Primary), 18 states (Auxiliary), and 25 states (Imputed) were used to segment the genome; these states were then grouped and colored to highlight the predicted functional elements. [file MOL2-16-750-s002.TIF]
