# Supplementary material for: Epigenetic readers and lung cancer: the rs2427964C>T variant of the bromodomain and extraterminal domain gene BRD3 is associated with poorer survival outcome in NSCLC
Source: Mol Oncol. 2021 Oct 15;16(3):750–63. doi: 10.1002/1878-0261.13109 (PMC8807359; doi:10.1002/1878-0261.13109)
Supplement: Supplementary file 4 — Table S1. Univariate analysis for OS by clinicopathologic features in the discovery and validation cohorts. Table S2. Summary of studied polymorphisms. Table S3. Six polymorphisms associated with OS in the discovery cohorts with P < 0.05 and their validation. Table S4. Summary of the functional annotation for five polymorphisms within LD. Table S5. Stratified analysis of the effects of rs2427964C>T genotypes under a recessive model on survival outcomes. [file MOL2-16-750-s004.docx]

| Supplementary Table1. Univariate analysis for overall survival by clinicopathologic features in the discovery and validation cohorts. | | | | | | | | | |
| --- | --- | --- | --- | --- | --- | --- | --- | --- | --- |
|  | Discovery set | | | |  | Validation set | | | |
| Variables | No. of  cases | No. of  death (%)^a^ | 5Y-OSR (%)^b^ | Log-Rank  *P* |  | No. of  Cases | No. of  death (%)^a^ | 5Y-OSR (%)^b^ | Log-Rank  *P* |
| Overall | 349 | 119 (34.1) | 56 |  |  | 424 | 85 (20.1) | 71 |  |
| Age (years) |  |  |  |  |  |  |  |  |  |
| < 64 | 169 | 47 (27.8) | 63 | 3 × 10^-3^ |  | 174 | 30 (17.2) | 76 | 0.18 |
| ≥ 64 | 180 | 72 (40.0) | 48 |  |  | 250 | 55 (22.0) | 67 |  |
| Gender |  |  |  |  |  |  |  |  |  |
| Male | 264 | 98 (37.1) | 54 | 0.15 |  | 301 | 71 (23.6) | 66 | 2 × 10^-3^ |
| Female | 85 | 21 (24.7) | 60 |  |  | 123 | 14 (11.4) | 81 |  |
| Smoking status |  |  |  |  |  |  |  |  |  |
| Never | 82 | 21 (25.6) | 64 | 0.20 |  | 148 | 19 (12.8) | 81 | 2 × 10^-3^ |
| Ever | 267 | 98 (36.7) | 53 |  |  | 276 | 66 (23.9) | 65 |  |
| Pack-years ^c^ |  |  |  |  |  |  |  |  |  |
| < 42 | 159 | 58 (36.5) | 54 | 0.24 |  | 170 | 35 (20.6) | 66 | 0.22 |
| ≥ 42 | 108 | 40 (37.0) | 54 |  |  | 106 | 31 (29.6) | 62 |  |
| Histological type |  |  |  |  |  |  |  |  |  |
| SCC | 178 | 59 (33.2) | 58 | 0.46 |  | 159 | 42 (26.4) | 65 | 0.03 |
| AC | 167 | 58 (34.7) | 52 |  |  | 253 | 39 (15.4) | 75 |  |
| LC | 4 | 2 (50.0) | 50 |  |  | 12 | 4 (33.3) | 63 |  |
| Pathologic stage |  |  |  |  |  |  |  |  |  |
| I | 186 | 44 (23.7) | 65 | 5 × 10^-5^ |  | 189 | 14 (7.41) | 91 | 2 × 10^-8^ |
| II-IIIA | 163 | 75 (46.0) | 45 |  |  | 235 | 71 (30.2) | 55 |  |
| Adjuvant therapy |  |  |  |  |  |  |  |  |  |
| No | 105 | 44 (41.9) | 48 | 0.57 |  | 73 | 26 (35.6) | 51 | 0.33 |
| Yes | 58 | 31 (53.5) | 41 |  |  | 162 | 45 (27.8) | 57 |  |
| Abbreviations: 5Y-OSR, 5 year-overall survival rate; SCC, squamous cell carcinoma ; AC, adenocarcinoma; LCC, large cell carcinoma  ^a^ Row percentage.  ^b^ Proportion of survival derived from Kaplan-Meier analysis.  ^c^ In ever-smokers. | | | | | | | | | |

| Supplementary Table 2. Summary of studied polymorphisms | | | | | | | | | |
| --- | --- | --- | --- | --- | --- | --- | --- | --- | --- |
| Polymorphisms^a^ | Affected Gene | Chromosome | Allele | Genotype | | | MAF | JPT | HWE-*P* |
|  |  |  |  | 11^b^ | 12^b^ | 22^c^ |  |  |  |
| rs2506711 | BRD3 | 9 | CT | 113 | 163 | 72 | 0.44 | 0.48 | 0.35 |
| rs2302107 | BRD2 | 17 | TC | 206 | 114 | 18 | 0.22 | 0.22 | 0.67 |
| rs298747 | BRD4 | 3 | CA | 137 | 153 | 38 | 0.35 | 0.37 | 0.63 |
| rs1537306 | BRD2 | 20 | GC | 301 | 39 | 0 | 0.06 | 0.17 | 0.26 |
| rs4792364 | BRD2 | 17 | CG | 141 | 164 | 31 | 0.34 | 0.22 | 0.09 |
| rs9358050 | BRD2 | 6 | CT | 216 | 106 | 18 | 0.21 | 0.21 | 0.30 |
| rs2810490 | BRD3 | 9 | AG | 124 | 150 | 62 | 0.41 | 0.48 | 0.17 |
| rs1075654 | BRD3 | 9 | GA | 268 | 67 | 4 | 0.11 | 0.17 | 0.93 |
| rs2520095 | BRD3 | 9 | GA | 157 | 135 | 45 | 0.33 | 0.42 | 0.07 |
| rs3752847 | BRD2 | 6 | CA | 207 | 111 | 18 | 0.22 | 0.23 | 0.54 |
| rs17795020 | BRD4 | 19 | CT | 92 | 153 | 92 | 0.50 | 0.47 | 0.09 |
| rs9245 | BRD2 | 1 | CA | 199 | 119 | 20 | 0.24 | 0.26 | 0.69 |
| rs7302973 | BRD2 | 12 | CT | 110 | 167 | 61 | 0.43 | 0.38 | 0.86 |
| rs12323861 | BRD2 | 14 | TC | 295 | 37 | 2 | 0.06 | 0.17 | 0.48 |
| rs4941403 | BRD2 | 13 | AG | 109 | 161 | 61 | 0.43 | 0.38 | 0.91 |
| rs17721902 | BRD4 | 19 | CA | 271 | 62 | 4 | 0.10 | 0.15 | 0.83 |
| rs411988 | BRD2 | 17 | GA | 213 | 113 | 14 | 0.21 | 0.26 | 0.84 |
| rs10417567 | BRD4 | 19 | CT | 257 | 77 | 4 | 0.13 | 0.14 | 0.51 |
| rs7138032 | BRD2 | 12 | TC | 81 | 177 | 80 | 0.50 | 0.44 | 0.38 |
| rs9525520 | BRD2 | 13 | TG | 106 | 177 | 57 | 0.43 | 0.44 | 0.24 |
| rs35417544 | BRD2 | 2 | TC | 208 | 112 | 18 | 0.22 | 0.24 | 0.57 |
| rs8112108 | BRD4 | 19 | CT | 161 | 134 | 29 | 0.30 | 0.33 | 0.88 |
| rs13164 | BRD2 | 22 | CG | 133 | 167 | 41 | 0.37 | 0.42 | 0.30 |
| rs3786853 | BRD4 | 19 | CT | 125 | 172 | 38 | 0.37 | 0.32 | 0.06 |
| rs1770341 | BRD2 | 6 | TC | 209 | 110 | 15 | 0.21 | 0.26 | 0.91 |
| rs3756661 | BRD2 | 5 | TG | 187 | 130 | 20 | 0.25 | 0.23 | 0.68 |
| rs35949820 | BRD3 | 9 | TG | 154 | 151 | 32 | 0.32 | 0.37 | 0.57 |
| rs3756663 | BRD2 | 5 | CA | 233 | 90 | 13 | 0.17 | 0.18 | 0.25 |
| rs16940820 | BRD2 | 17 | AC | 192 | 125 | 18 | 0.24 | 0.26 | 0.69 |
| rs4141885 | BRD2 | 6 | TA | 186 | 123 | 28 | 0.27 | 0.19 | 0.24 |
| rs6941421 | BRD2 | 6 | TC | 140 | 157 | 39 | 0.35 | 0.30 | 0.62 |
| rs9393790 | BRD2 | 6 | GT | 200 | 116 | 19 | 0.23 | 0.33 | 0.69 |
| rs10993889 | BRD3 | 9 | AG | 143 | 160 | 37 | 0.34 | 0.33 | 0.43 |
| rs12186358 | BRD2 | 5 | TC | 198 | 127 | 15 | 0.23 | 0.27 | 0.34 |
| rs12597095 | BRD2 | 16 | GA | 259 | 75 | 6 | 0.13 | 0.12 | 0.83 |
| rs2079014 | BRD4 | 19 | TC | 154 | 151 | 35 | 0.33 | 0.35 | 0.82 |
| rs2270114 | BRD2 | 17 | GC | 135 | 148 | 62 | 0.39 | 0.36 | 0.06 |
| rs238237 | BRD2 | 17 | GA | 106 | 173 | 63 | 0.44 | 0.49 | 0.61 |
| rs463271 | BRD2 | 22 | CT | 171 | 133 | 31 | 0.29 | 0.30 | 0.49 |
| rs9532878 | BRD2 | 13 | TC | 145 | 153 | 42 | 0.35 | 0.38 | 0.87 |
| rs1108613 | BRD2 | 14 | CT | 224 | 108 | 6 | 0.18 | 0.16 | 0.08 |
| rs13347026 | BRD4 | 19 | TC | 292 | 41 | 3 | 0.07 | 0.13 | 0.26 |
| rs17448937 | BRD2 | 13 | CG | 287 | 47 | 0 | 0.07 | 0.11 | 0.17 |
| rs17581597 | BRD2 | 1 | CT | 162 | 137 | 37 | 0.31 | 0.31 | 0.33 |
| rs218495 | BRD2 | 3 | TC | 175 | 130 | 31 | 0.29 | 0.36 | 0.34 |
| rs2645479 | BRD2 | 17 | GA | 160 | 142 | 34 | 0.31 | 0.37 | 0.76 |
| rs3200401 | BRD2 | 11 | CT | 246 | 83 | 8 | 0.15 | 0.24 | 0.75 |
| rs4740123 | BRD2 | 8 | GC | 124 | 167 | 45 | 0.38 | 0.38 | 0.34 |
| rs6018427 | BRD2 | 20 | AG | 231 | 98 | 10 | 0.17 | 0.14 | 0.92 |
| rs6125196 | BRD2 | 20 | TC | 215 | 110 | 11 | 0.20 | 0.12 | 0.50 |
| rs769040 | BRD2 | 13 | CG | 259 | 73 | 5 | 0.12 | 0.13 | 0.96 |
| rs8001004 | BRD2 | 13 | TC | 94 | 159 | 79 | 0.48 | 0.45 | 0.46 |
| rs8070007 | BRD2 | 17 | AG | 164 | 151 | 23 | 0.29 | 0.19 | 0.13 |
| rs10417780 | BRD4 | 19 | GA | 143 | 147 | 39 | 0.34 | 0.28 | 0.90 |
| rs10739575 | BRD2 | 9 | AG | 122 | 160 | 48 | 0.39 | 0.42 | 0.70 |
| rs16948010 | BRD2 | 17 | TC | 229 | 95 | 9 | 0.17 | 0.16 | 0.82 |
| rs1471560 | BRD2 | 8 | CA | 81 | 171 | 70 | 0.48 | 0.41 | 0.26 |
| rs3195676 | BRD2 | 5 | CT | 112 | 153 | 52 | 0.41 | 0.38 | 0.98 |
| rs12366 | BRD2 | 2 | GT | 127 | 110 | 27 | 0.31 | 0.39 | 0.66 |
| Abbreviation: MAF, minor allele frequency; JPT, Japanese in Tokyo; HWE-*P*, *P* value for Hardy-Weinberg equilibrium  ^a^ Eighteen polymorphisms with genotype failure or deviations from HWE were excluded.  ^b^ 1 represents the wild allele and 2 represents the mutant allele. | | | | | | | | | |

| Supplementary Table 3. Six polymorphisms associated with overall survival in the discovery cohorts with *P*^a^ < 0.05 and their validation. | | | | | | | | |
| --- | --- | --- | --- | --- | --- | --- | --- | --- |
|  | Discovery cohort (n=349) | | | |  | Validation cohort (n=424) | | |
| Polymorphisms |  | Dominant | Recessive | Codominant |  | Dominant | Recessive | Codominant |
| rs2506711C>T |  | 0.19 | 0.01 | 0.02 |  | 0.45 | 0.01 | 0.05 |
| rs2302107T>C |  | 0.01 | 0.07 | 0.00 |  | 0.42 | 0.29 | 0.29 |
| rs298747C>A |  | 0.02 | 0.15 | 0.01 |  | 0.20 | 0.51 | 0.56 |
| rs1537306G>C |  | 0.01 | - | 0.01 |  | 0.60 | 0.05 | 0.34 |
| rs4792364C>G |  | 0.51 | 0.02 | 0.10 |  | 0.19 | 0.90 | 0.37 |
| rs9358050C>T |  | 0.05 | 0.85 | 0.10 |  | 0.21 | 0.47 | 0.20 |
| ^a^*P* values were calculated using multivariate Cox proportional hazard models, adjusted for age, gender, smoking status, tumor histology, adjuvant therapy, and pathologic stage. | | | | | | | | |

| Supplementary Table 4. Summary of the functional annotation for five polymorphisms within linkage disequilibrium | | | | | | | |
| --- | --- | --- | --- | --- | --- | --- | --- |
| rs number | Gene | Position | Chromatin state^a^ | Promoter histone marks | Enhancer histone marks | Motifs changed | Protein binding  (A549) |
| rs2810492 | BRD3  (intronic) | 134058850 | Enhancers(66),  Strong transcription(52), Active TSS(3) | 2 tissues | 21 tissues | NF-I, Rad21, SRF | CTCF, CFOS |
|  |  |  |  |  |  |  |  |
| rs1076148 | BRD3  (intronic) | 134062535 | Enhancers(72),  Strong transcription(44), Active TSS(4) |  |  | PTF1-beta |  |
|  |  |  |  |  |  |  |  |
| rs2427964 | BRD3  (intronic) | 134066835 | Active TSS(125),  Strong transcription(1), Bivalent/Poised TSS(1) | 24 tissues |  | Foxp1,Zfp105 | SIN3A, ETS-1 |
|  |  |  |  |  |  |  |  |
| rs2506711 | BRD3  (3.9kb 5' UTR) | 134072482 | Repressed PolyComb(88), Quiescent/Low(35), Heterochromatin(4) |  |  | Ascl2,Lmo2-complex |  |
|  |  |  |  |  |  |  |  |
| rs2905063 | BRD3  (8.2kb 5' UTR) | 134076724 | Repressed PolyComb(33), Quiescent/Low(35), Heterochromatin(59) |  |  | RXRA |  |
| Abbreviations: TSS, Transcription Start Site  In-silico anlaysis using ENCODE-based web-tools rSNPBase(now rVarBase), HaploReg.  ^a^Results of chromatin data (No. of chromatin data) | | | | | | | |

| Supplementary Table 5. Stratified analysis of the effects of rs2427964C>T genotypes under a recessive model on survival outcomes | | | |
| --- | --- | --- | --- |
|  | Overall survival | | |
| Variables | HR(95% CI)^a^ | *P*^a^ | *P*_H_ |
| Age (years) |  |  |  |
| < 64 | 2.08 (1.22-3.53) | 0.01 | 0.70 |
| ≥ 64 | 1.82 (1.20-2.75) | 0.01 |  |
| Gender |  |  |  |
| Male | 1.92 (1.34-2.74) | 3 × 10^-4^ | 0.83 |
| Female | 1.77 (0.79-3.96) | 0.16 |  |
| Smoking status |  |  |  |
| Never | 2.02 (0.96-4.27) | 0.06 | 0.90 |
| Ever | 1.92 (1.33-2.75) | 0.001 |  |
| Histological type |  |  |  |
| SCC | 1.35 (0.81-2.25) | 0.25 | 0.04 |
| AC | 2.78 (1.77-4.35) | 8 × 10^-6^ |  |
| Pathologic stage |  |  |  |
| I | 2.15 (1.18-3.93) | 0.01 | 0.63 |
| II+ IIIA | 1.81 (1.23-2.66) | 1 × 10^-3^ |  |
| Abbreviations: HR, hazard ratio; CI, confidence interval; *P_H_*, *P*-value of test for homogeneity; SCC, squamous cell carcinoma; AC, adenocarcinoma  ^a^ HRs, 95% CIs and corresponding *P*-values were calculated using multivariate Cox proportional hazard models, adjusted for other variables. | | | |
